# Editorial: Cognitive and emotional responses towards diabetes among socio-demographically diverse populations

**DOI:** 10.3389/fpsyg.2024.1452892

**Published:** 2024-08-06

**Authors:** Shiri Shinan-Altman, Lee Greenblatt-Kimron, Hanneke J. A. Smaling

**Affiliations:** ^1^Louis and Gabi Weisfeld School of Social Work, Bar-Ilan University, Ramat Gan, Israel; ^2^School of Social Work, Ariel University, Ariel, Israel; ^3^Department of Public Health and Primary Care, Leiden University Medical Center, Leiden, Netherlands

**Keywords:** cognitive responses, emotional responses, diabetes, diverse populations, disease management (DM)

The field of diabetes care increasingly recognizes the essential roles of cognitive and emotional factors in disease management. This editorial includes three studies aimed to collectively broaden the understanding of these psychosocial dimensions across diverse populations.

“*The relationship between emotional self-awareness, emotion regulation, and diabetes distress among Italian and Dutch adults with type 1 diabetes”* (Bassi et al.) shows links between emotional self-awareness and diabetes distress. This study underscores the significant, negative association between clarity of feelings and diabetes distress. Moreover, it advocates emotional self-awareness as a vital component in psychological interventions, focused on alleviating distress in diabetes management.

“*Time and risk preferences and the perceived effectiveness of incentives to comply with diabetic retinopathy screening among older adults with type 2 diabetes”* (Tang et al.) explores how behavioral economic principles such as time and risk preferences influence compliance with diabetic retinopathy screening in rural and urban areas of Guangdong, southern China. This research suggests how personalized incentives could be structured to improve adherence to recommended screenings. In this way, the study highlights ways to prevent severe complications in diabetes management.

“*The mediating role of diabetes stigma and self-efficacy in relieving diabetes distress among patients with type 2 diabetes mellitus: a multicenter cross-sectional study”* (Xing et al.) addresses the impact of social support, diabetes stigma, and self-efficacy on managing diabetes distress in Hainan Province. The findings highlight that reducing diabetes stigma and enhancing self-efficacy can significantly alleviate distress, highlighting targeted areas for psychosocial interventions.

The three studies create a picture that illustrates the complex associations between cognitive-emotional factors and diabetes care across various contexts. Together, these studies emphasize the importance of psychosocial dimensions in diabetes management. Further, they underscore the necessity of integrating psychological interventions into standard care protocols with the aim of optimizing health outcomes in diabetic populations.

## Author contributions

SS-A: Writing – original draft. LG-K: Writing – review & editing. HS: Writing – review & editing.

